# Therapeutic application of feeding strategies in chronic pancreatitis: the potential for jejunal feeding and elemental diet to modulate pancreatic secretion to alleviate painful symptoms

**DOI:** 10.3389/fphys.2026.1828606

**Published:** 2026-07-17

**Authors:** Mei Cai, Niwen Kong, Baraa Abdelghne, Anna Evans Phillips, Sohail Z. Husain, Stephen J. Pandol, Yi Jiang

**Affiliations:** 1 Department of Internal Medicine, Scripps Clinic/Scripps Green Hospital, La Jolla, CA, United States; 2 Division of Gastroenterology and Hepatology, Stanford University, Stanford, CA, United States; 3 School of Humanities and Sciences, Stanford University, Stanford, CA, United States; 4 Division of Gastroenterology, Hepatology, Nutrition, University of Pittsburgh School of Medicine, Pittsburgh, PA, United States; 5 Division of Gastroenterology, Hepatology, and Nutrition, Department of Pediatrics, Stanford University School of Medicine, Stanford, CA, United States; 6 Karsh Division of Gastroenterology, Departments of Medicine, Cedars-Sinai Medical Center, Los Angeles, CA, United States

**Keywords:** chronic pancreatitis, elemental diet, jejunal feeding, nutritional strategies, pain phenotype, therapeutic feeding

## Abstract

**Background:**

Chronic pancreatitis (CP) often leaves patients with debilitating abdominal pain, postprandial symptom exacerbation, and progressive malnutrition. While traditional management focuses on analgesics and enzyme replacement, strategies aimed to reduce pancreatic secretory stimulation may offer an adjunct to standard approaches.

**Aim:**

We explore the physiologic rationale and the existing clinical evidence for jejunal feeding and elemental diets to attenuate pancreatic secretion and improve the symptom burden of CP.

**Methods:**

We summarize pancreatic exocrine regulation, emphasizing how nutrient composition and intestinal delivery site shape feedback pathways and pancreatic secretion. We review the physiologic and observational studies of mid-to-distal jejunal tube feeding and elemental diets in animal models, healthy individuals, and patients with CP pain.

**Results:**

Studies suggest that jejunal feeding can reduce pancreatic stimulation and may be associated with significant reductions in abdominal pain and analgesic utilization, with improvements in nutritional status. Furthermore, elemental diets, composed of free amino acids, medium-chain triglycerides, and simple carbohydrates, may complement this effect by facilitating efficient nutrient absorption while keeping the pancreas relatively quiescent. However, evidence remains limited by heterogeneity and predominantly nonrandomized study designs.

**Conclusions:**

Jejunal feeding and elemental diets represent potential adjunctive strategies for selected CP patients with pain-predominant phenotypes and postprandial symptom exacerbation. Prospective trials are needed to define patient selection, optimal formula, delivery location, durability of benefit, and standardized outcomes regarding patient-reported pain impact, nutritional markers, and treatment-related complications.

## 1 Introduction

Chronic pancreatitis (CP) is a progressive fibroinflammatory disease characterized by recurrent pancreatic parenchymal damage leading to structural and functional impairment associated with significant abdominal pain symptoms (Hines and Pandol, 2024). Epidemiological studies estimate a prevalence of 43 to 72 per 100,000 adults and an increase in incidence over the past few decades (Bampton et al., 2025; Cai et al., 2023; Hirota et al., 2012; Machicado et al., 2019; Sellers et al., 2018; Thierens et al., 2025; Yadav et al., 2011). The chronic, escalating nature of CP burdens both patients and the healthcare system, requiring longitudinal management of acute-on-chronic pain, long-term symptom mitigation, and surveillance for disease-related complications (Hines and Pandol, 2024; Yadav et al., 2023). Recurrent pain flares often prompt repeat diagnostic evaluations and hospitalizations. These episodes often require advanced imaging modalities, including computed tomography (CT), as well as procedures such as endoscopic retrograde cholangiopancreatography (ERCP), which further increase healthcare utilization, procedural risks, and overall patient burden (Poulsen et al., 2013).

CP is a complex disorder with diverse etiologies, variable clinical trajectories, and a broad spectrum of complications (Hines and Pandol, 2024; Thierens et al., 2025). While some patients follow an indolent course progressing toward exocrine and endocrine insufficiency, over 80% have debilitating chronic abdominal pain (Goodarzi et al., 2021; Hart et al., 2016; Kempeneers et al., 2021; Thierens et al., 2025; Vipperla et al., 2021). This pain is commonly reported to be exacerbated by food intake, leading to food avoidance, malnutrition, and functional decline (Goulden, 2013; Khurmatullina et al., 2025). Concurrent gastrointestinal (GI) symptoms – such as nausea, vomiting, early satiety, and delayed transit – are also common (Goulden, 2013). Opioid analgesics, frequently used for pain management, can cause narcotic bowel syndrome and further exacerbate GI dysmotility; this creates a vicious cycle where worsening dysmotility triggers more pain and discomfort, leading to increased narcotic reliance (Goulden, 2013; Poulsen et al., 2013). Thus, alternative therapeutic strategies aimed to reduce CP-associated postprandial pain may reduce narcotic dependence, improve nutritional intake, and enhance overall quality of life.

Genetic variants affecting trypsin regulation, ductal secretion, and inflammatory pathways (most notably *PRSS1, SPINK1, CTRC*, and *CFTR*) interact with anatomic variants and environmental exposures (such as alcohol and tobacco use) to shape the onset, severity, progression, and treatment response of CP-associated pain (Bertin et al., 2012; Go et al., 2005; Hines and Pandol, 2024; Hu et al., 2017; Rosendahl et al., 2008; Schneider et al., 2004). Persistent inflammation induces peripheral nociception, while neurogenic inflammation increases vascular permeability, edema, and further nerve activation. Structural abnormalities such as ductal strictures, intraductal stones, fibrosis, and pseudocysts may exacerbate pain through several overlapping mechanisms. These include impaired pancreatic ductal outflow with increased intraductal or intrapancreatic pressure, local inflammation and ischemia, and mass effect or compression of adjacent tissues by pseudocysts or inflammatory enlargement (Ahmed Ali et al., 2015; Beyer et al., 2020; Lin et al., 2025; A. Singh et al., 2023). These structural changes may also promote perineural inflammation and peripheral nociceptive signaling. Over time, sustained nociceptive input can induce central sensitization, heightening central nervous system (CNS) excitability and amplifying pain perception independent of ongoing tissue injury (Lin et al., 2025). Altogether, local inflammation, neuropathic remodeling, and CNS priming create a self-reinforcing pain state in CP (Lin et al., 2025). This complex genophenotypic framework contributes to heterogeneous pain experiences and inconsistent therapeutic efficacy, highlighting the need for more personalized, mechanism-based approaches to pain management in CP (Drewes et al., 2017; Goulden, 2013; Poulsen et al., 2013; Yadav et al., 2023).

Despite a range of therapeutics, persistent symptoms can continue to plague patients. Dietary counseling is commonly advised, and pancreatic enzyme replacement therapy (PERT) is indicated for exocrine pancreatic insufficiency to improve maldigestion, steatorrhea, and malnutrition, though it has not been shown to relieve CP-related pain in adults (Domínguez-Muñoz and Phillips, 2018; Gardner et al., 2020). Analgesic management ranging from non-opioid analgesics, to neuromodulators, to opioids, is used with inconsistent efficacy. For medically refractory pain, procedural interventions – including endoscopic interventions, celiac plexus block, and surgical procedures– may be pursued in select patients; however, only about 50-70% of patients experience meaningful pain improvement with these more advanced options. These approaches can require frequent interventions (such as prolonged stenting), and the durability of benefit may be limited, especially with less invasive options such as celiac plexus block, where pain relief can wane in a few months (Chan et al., 2025; Hines and Pandol, 2024; Issa et al., 2020; Machicado et al., 2025; van Veldhuisen et al., 2025). In addition, invasive procedures can be distressing for patients, as they often require significant time, expense, and physical stress, especially when pain persists or returns despite these efforts.

In contrast, nutritional therapy may represent a lower-risk adjunctive strategy in selected CP patients. Nutritional interventions are generally feasible, well-studied, and tolerated in other inflammatory GI disorders, such as eosinophilic esophagitis and inflammatory bowel disease (IBD) (Li Voti et al., 2025; Reed et al., 2017). Physiologically, pancreatic exocrine secretion is strongly influenced by intact nutrient exposure in the upper GI tract, and it is our understanding that the release of enzyme-rich secretions into an inflamed, fibrotic pancreas can exacerbate the pain seen in CP. Accordingly, attenuating pancreatic stimulation and exocrine secretion by modifying the route and composition of nutrient delivery may offer a potential strategy to reduce symptom burden while maintaining nutritional support. In this review, we explore the rationale and available evidence supporting this unconventional yet promising strategy, discuss its limitations, and outline key next steps for future investigation.

### 1.1 Literature search strategy

A literature search was conducted primarily in PubMed to identify articles relevant to chronic pancreatitis, pain management, enteral nutrition, jejunal feeding, elemental diets, and pancreatic exocrine secretion. Search terms included combinations of keywords and MeSH terms such as “chronic pancreatitis,” “pain,” “enteral nutrition,” “jejunal feeding,” “elemental diet,” and “pancreatic secretion.” Additional terms included “cholecystokinin,” “pancreatic exocrine function,” and “patient-reported symptoms.” References cited within relevant primary studies and review articles were also manually screened to identify foundational, physiologic, and historically significant publications that may not have been captured in the initial search. Articles were selected based on their relevance to the scope of this narrative review, with priority given to clinical studies, case series, physiologic studies of pancreatic secretion, and articles addressing enteral feeding strategies, nutrient-stimulated pancreatic secretion, or symptom outcomes in chronic pancreatitis. A summary of the primary studies informing this review is provided in **Table 1**.

**Table 1:** Studies examining the effects of feeding location and formula composition on pancreatic stimulation and clinical outcomes.

Study	Model/population	Intervention/comparison	Main relevant findings
Cassim and Allardyce, 1974	Animal models	Proximal jejunal feeding	Proximal jejunal feeding induced brisk secretion of bicarbonate-rich, enzyme-poor pancreatic fluid.
Vidon et al., 1978	Healthy human subjects (N = 17)	ED vs crushed food homogenate	Elemental diet resulted in lower lipase secretion compared with crushed food homogenate at comparable calorie delivery. Fasting baseline was not assessed.
Watanabe et al., 1986	Healthy human subjects (N = 9)	Increasing infusion rates of amino acid-based ED	Increasing elemental diet infusion rates produced dose-dependent increases in circulating CCK as well as pancreatic protein and bicarbonate secretion.
Vu et al., 1999	Healthy human subjects (n=8)	Feeding delivered to proximal jejunum just distal to the LoT vs distal jejunum (approx. 60 cm further)	Proximal jejunal feeding significantly increased pancreatic enzyme and bilirubin output above basal levels. Distal jejunal feeding did not significantly increase pancreatic enzyme secretion above baseline.
Symersky et al., 2002	Healthy human subjects (N = 8)	Medium-chain triglycerides vs long-chain triglycerides	Medium-chain triglycerides infusion did not cause any significant change from fasting baseline. Long-chain triglycerides infusion caused significant increases of lipase, amylase, bilirubin, CCK release, and gallbladder contraction.
Shea et al., 2003	Healthy human subjects (N = 6)	ED vs polymeric formula vs high-fat meal	ED increased CCK levels modestly above baseline. Polymeric formula and a high-fat meal increased CCK levels significantly more above baseline.
	Patients with CP (N = 8)	Supplemental oral ED	Median pain scores improved by 68.5% from baseline to study conclusion (p=0.011), with improved pain control in 6 of 8 patients and decreased narcotic use among responders.
O’Keefe et al., 2003	Healthy human subjects (N = 27)	Placebo (saline) vs oral vs duodenal vs IV nutrition	Intravenous nutrition produced pancreatic secretion levels comparable to placebo saline. Oral and duodenal complex meals markedly increased pancreatic enzyme secretion.
	Healthy human subjects (N = 10)	ED vs Polymeric meal	ED did not significantly increase amylase or trypsin above baseline. Polymeric meal increased amylase 6.2-fold and trypsin 3.5-fold above baseline ED increased lipase 7-fold above baseline. Polymeric meal increased lipase 11.4-fold above baseline
Kaushik et al., 2005	Healthy human subjects (N = 36)	Fasting vs intravenous nutrition vs duodenal feeding vs mid/distal jejunal feeding (40–60 cm distal to LoT)	Mid/distal jejunal feeding produced pancreatic enzyme secretion levels comparable to fasting and intravenous nutrition, and significantly lower than duodenal feeding.
	Healthy human subjects (n=13)	Duodenal polymeric diet vs duodenal ED	Duodenal ED produced less pancreatic stimulation than polymeric diet. Trypsin: ED increased trypsin 2.5-fold above baseline. Polymeric diet increased trypsin 3.5-fold above baseline. Lipase: ED did not significantly increase lipase above fasting baseline. Polymeric diet increased lipase 10-fold above baseline.
Stanga et al., 2005	Patients with CP (n=57)	Jejunal feeding via PEG-J or DPEJ	Persistent abdominal pain decreased from 96% to 23% over 180 days. Requirement for pain medication decreased from 91% to 27%.
Moore et al., 2006	Patients with CP (N = 25)	Jejunal feeding via various routes	45% achieved near-complete or complete pain resolution over a mean duration of 4.6 months (p=0.008).
Skipworth et al., 2011	Patients with CP (n=58)	Nasojejunal feeding	79.3% of patients reported resolution of abdominal pain and cessation of opioid analgesics over a median duration of 47 days
Kataoka et al., 2014	Patients with CP (n=596)	Supplemental oral low-fat elemental diet for 12 weeks	Visual analog pain scores decreased by 32.9 mm from a baseline of 52.9 mm (p<0.001).

LoT, ligament of Treitz; ED, elemental diet; PEG-J, percutaneous endoscopic gastrojejunostomy; DPEJ, direct percutaneous endoscopic jejunostomy; CCK, cholecystokinin; CP, chronic pancreatitis.

## 2 Molecular and physiologic overview of pancreatic secretory response

### 2.1 Mechanisms of nutrition-stimulated exocrine secretion and review of anatomy

Nutrient-stimulated pancreatic exocrine secretion is governed by integrated neurohormonal control, in which the intestinal hormones, cholecystokinin (CCK), and secretin act in concert with vagal-cholinergic pathways (Hall and Hall, 2021; Owyang and Logsdon, 2004; Pandol, 2011; Taylor et al., 2015). Postprandially, partially digested chyme enters the duodenum. Luminal peptides and fatty acids stimulate specialized enteroendocrine cells, also known as duodenal I cells, on their apical surface, triggering basolateral release of CCK into circulation. Simultaneously, luminal acidity stimulates duodenal S cells to secrete secretin into the bloodstream. Luminal acid is the dominant physiologic stimulus for secretin release; however, additional luminal factors have been shown under experimental conditions to stimulate immunoreactive secretin, suggesting that secretin regulation is multifactorial rather than exclusively acid-dependent (Bondesen et al., 1985; Hanssen, 1980). CCK- and secretin-mediated signaling is amplified by vagovagal reflexes, which enhance cholinergic output to the pancreas. CCK acts on pancreatic acinar cells to stimulate exocytosis of digestive enzymes and also promotes gallbladder contraction for bile release. Secretin acts on pancreatic ductal epithelial cells to secrete a bicarbonate-rich fluid, which neutralizes gastric acid and raises intraluminal pH. The more alkaline environment optimizes the activity of the brush border enzyme enteropeptidase, enabling conversion of the proenzyme, or zymogen, trypsinogen to trypsin, and subsequent activation of other pancreatic zymogens, kickstarting intraluminal digestion (Lu et al., 2021; Owyang and Logsdon, 2004; Pandol, 2011; Taylor et al., 2015).

The anatomical features of the small intestine are contextually important to their contribution to pancreatic secretion. The duodenum starts after the pylorus and extends approximately 20–30 centimeters (cm). The jejunum begins at the ligament of Treitz (LoT) and stretches roughly 200 cm in length; the proximal jejunum is characterized by a thick wall, wide lumen, rich vascular supply, and prominent mucosal folds, making it a major site of nutrient absorption. Progressing distally, the jejunal intestinal wall gradually becomes thinner, with less vascularity, and shorter villi. The transition from proximal jejunum to distal jejunum to the start of the ileum is gradual and often indistinct. The ileum is approximately 300 cm in length, prior to its termination at the ileocecal valve (Dalley et al., 2023; Hall and Hall, 2021; Power et al., 2025).

### 2.2 Intestinal site of delivery on pancreatic secretion

**Figure 1:**
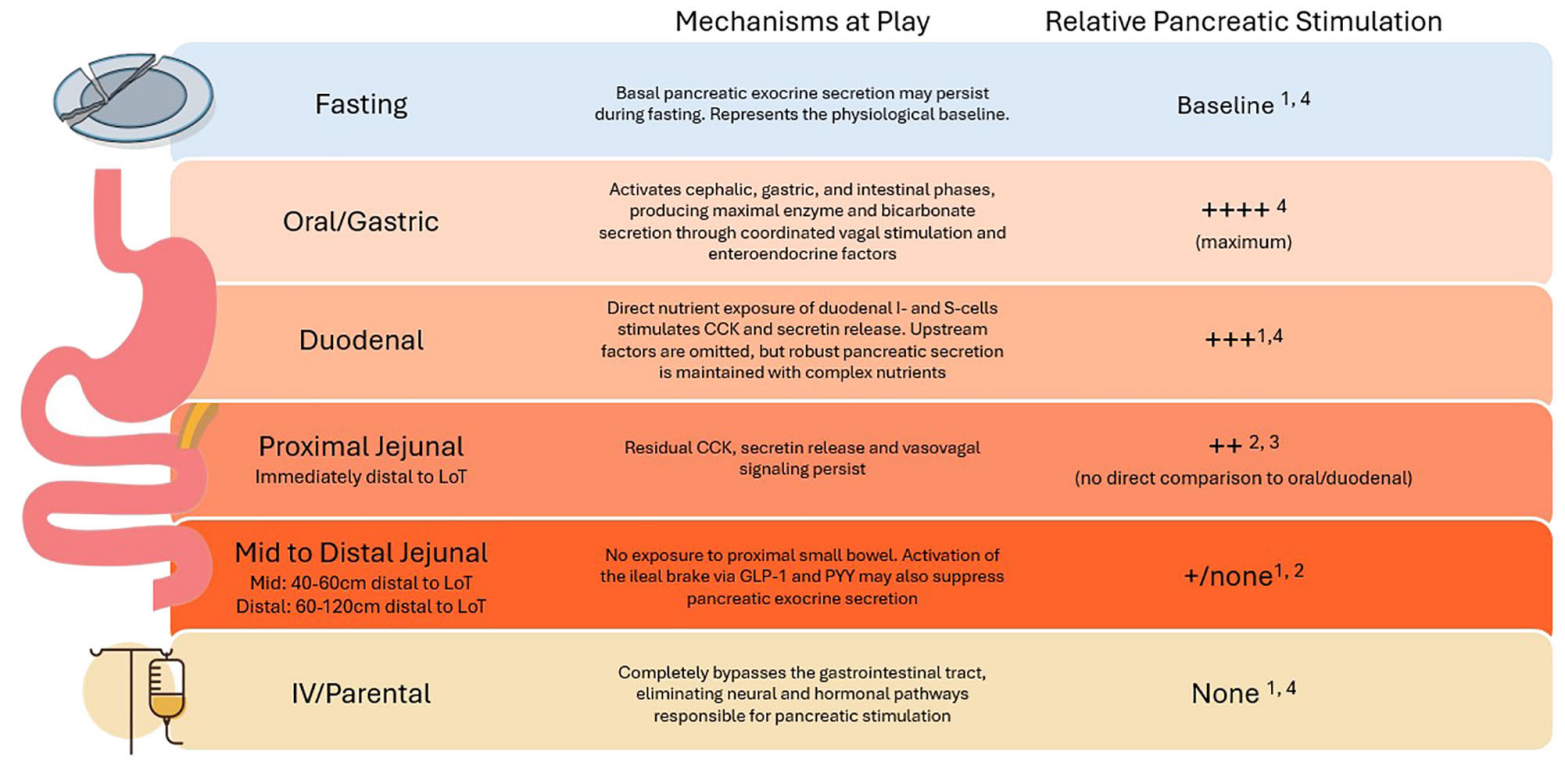
Pancreatic exocrine stimulation by site of nutrient delivery. Pancreatic exocrine secretion (lipase, trypsin, amylase, bicarbonate) and hormonal responses (cholecystokinin (CCK) and secretin) vary depending on the site of nutrient administration. No single study directly compares pancreatic stimulation across all feeding sites. This image synthesizes data from multiple studies (^1^Kaushik et al. (2005), ^2^Vu et al. (1999), ^3^Cassim and Allardyce (1974), ^4^O’Keefe et al. (2003)) to illustrate the relative potency of different intestinal locations. Although each study measured responses at varying sites, the available data allow limited extrapolation to compare effects across feeding locations. Some graphical elements were created with BioRender.com and modified by the authors. LoT, ligament of Treitz, GLP-1, glucagon-like peptide-1, PYY, peptide YY.

The magnitude of pancreatic exocrine secretion varies depending on the intestinal site of nutrient delivery. Oral and gastric feeding initiate the strongest pancreatic response, combining the effects of cephalic-phase mechanisms (mastication, gastric distension, and vagovagal cholinergic activation) with downstream stimuli. Following gastric emptying, the duodenum serves as the initial site of intestinal stimulation and is the first site exposed to acidic chyme, fatty acids, and proteins (Hall and Hall, 2021; Lu et al., 2021). These stimuli activate S and I cells (previously described in Section IIIA), which are most abundant in the proximal small intestine and taper distally (Roberts et al., 2019; Sjölund et al., 1983). This physiology is well established, and duodenal nutrient delivery reliably produces marked increases in plasma CCK, pancreatic enzyme and bicarbonate output as compared to fasting or saline placebo (Kaushik et al., 2005; O’Keefe et al., 2003). These studies are highlighted here to establish the duodenum as a physiologic reference point for intestinal nutrient stimulation. In contrast to fasting, parenteral nutrition, or more distal jejunal feeding, duodenal nutrition delivery consistently produces a robust exocrine response. This reference point is important when interpreting studies of more distal nutrient delivery, where pancreatic stimulation progressively diminishes. Relative pancreatic exocrine stimulation is also summarized in **Figure 1**.

Immediately distal to the LoT, the proximal jejunum appears to retain a modest capacity to stimulate pancreatic secretion. Animal models demonstrated brisk secretion of bicarbonate-rich but relatively enzyme-poor pancreatic fluid in response to proximal jejunal feeding (Cassim and Allardyce, 1974). In human studies, proximal jejunal nutrient delivery increased lipase, trypsin, and amylase secretion (Vu et al., 1999). Important to note, these studies did not directly compare proximal jejunal feeding with oral or duodenal delivery, limiting conclusions regarding its relative potency.

In contrast, mid-to-distal jejunal feeding produces a largely quiescent pancreatic exocrine response. Of note, across these studies, definitions of “proximal,” “mid,” and “distal” small-bowel delivery vary; distances and anatomical landmarks are reported as defined by the authors. Mid-jejunal (40–60 cm distal to LoT) and distal jejunal (100–120 cm distal to LoT) feeding resulted in trypsin levels marginally above baseline (without statistical significance), and levels significantly lower than those observed with duodenal feeding (Kaushik et al., 2005). Similarly, distal jejunal feeding (defined as 60 cm distal to LoT) did not significantly increase lipase, trypsin, or amylase secretion above fasting baseline (Vu et al., 1999).

Parenteral or intravenous (IV) nutrition bypasses luminal sensing altogether, and does not seem to elicit stimulation of pancreatic exocrine function. Parenteral feeding does not produce significant increases in enzyme secretion or hormonal responses compared with fasting conditions (Kaushik et al., 2005; O’Keefe et al., 2003). However, while parenteral nutrition can achieve pancreatic “rest”, its associated limitations and complications often lead clinicians to favor enteral feeding when feasible.

### 2.3 Composition of feeding on pancreatic stimulation

Elemental diets (ED) consist of nutrients in their most basic forms, and include free amino acids, medium-chain triglycerides (MCTs), and simple carbohydrates. Because these nutrients require minimal digestive processing prior to absorption, ED are hypothesized to reduce pancreatic exocrine stimulation compared with polymeric meals. To better understand this rationale, it is helpful to review studies that examine how the form and quantity of individual macronutrients influence pancreatic exocrine secretion.

Protein quantity appears to be a major determinant of pancreatic exocrine output. In a human study, Vidon et al. demonstrated that pancreatic enzyme secretion correlated more closely with nitrogen delivery than with caloric load alone, supporting the concept that protein content is one of the primary drivers of enzyme output (Vidon et al., 1978). Experimental studies support this relationship. In canines, Wolfe et al. showed that duodenal perfusion of free amino acids elicited pancreatic secretory responses comparable to those observed in perfusion of an ED, suggesting that the pancreatic stimulatory effects of ED are largely attributable to their amino acid content (Wolfe et al., 1975). Consistent with this, Watanabe et al. demonstrated a dose-response relationship in humans: increasing amino acid-based elemental infusion led to a stepwise rise in circulating CCK, accompanied by increased pancreatic protein and bicarbonate secretion, supporting a CCK-mediated mechanism linking amino acid quantity to pancreatic stimulation (Watanabe et al., 1986).

Dietary fat composition also plays an important role in pancreatic exocrine secretion. In healthy adults, Symersky et al. demonstrated intrajejunal infusion of MCTs failed to significantly stimulate pancreatic enzyme secretion above baseline, whereas isocaloric long-chain triglycerides (LCTs) infusion elicited a significant response with increase in CCK, pancreatic lipase and amylase secretion, bilirubin output, and gallbladder contraction (Symersky et al., 2002). These findings are consistent with known fat digestion physiology: MCTs do not require emulsification or micelle formation and can be absorbed directly into the portal circulation, whereas LCTs must strongly activate pancreatic and biliary pathways for effective digestion and absorption (Hall and Hall, 2021).

In contrast to the clear stimulatory effects with proteins and LCTs, the role of carbohydrates in pancreatic exocrine secretion appears limited. Available studies suggest carbohydrates are weak stimulators of pancreatic secretion and may even exert inhibitory effects. Vidon et al. reported greater pancreatic enzyme output with a crushed food homogenate (with less carbohydrate content) than with elemental formula, though interpretation is limited given the presence of intact proteins and lipids in the crushed food homogenate (Vidon et al., 1978). Several studies have demonstrated that the administration of an isotonic sugar solution has minimal effects on CCK, pancreatic protein, or bicarbonate secretion (Lawrence et al., 1961; Ragins et al., 1973; Schapiro et al., 1967; Watanabe et al., 1986). A clinical study by Dyck et al. showed intrajejunal glucose suppresses pancreatic volume and bicarbonate secretion (Dyck, 1971). Overall, carbohydrates appear to play a minor (or occasionally even an inhibitory) role in pancreatic exocrine regulation compared with proteins and lipids.

**Figure 2:**
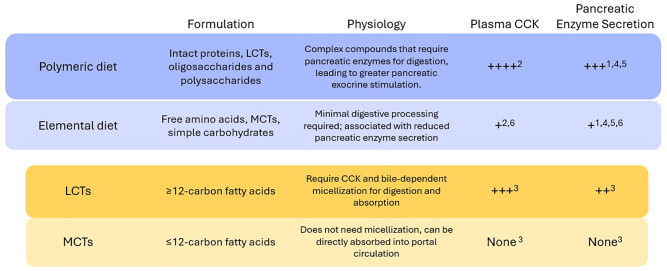
Effects of diet composition on cholecystokinin (CCK) release and pancreatic enzyme secretion. The image summarizes the reported effects of polymeric diets, elemental diets, long-chain triglycerides (LCTs), and medium-chain triglycerides (MCTs) on circulating cholecystokinin (CCK) levels and intra-luminal pancreatic enzyme secretion, as synthesized from multiple studies (^1^Vidon et al. (1978), ^2^Shea et al. (2003), ^3^Symersky et al. (2002), ^4^Kaushik et al. (2005), ^5^O’Keefe et al. (2003), ^6^Watanabe et al. (1986)). In general, polymeric diets and LCTs are associated with greater stimulation of CCK release and pancreatic enzyme secretion, whereas elemental diets and MCTs demonstrate reduced or no stimulation. The image is intended to illustrate overall trends, as the underlying studies are heterogeneous with respect to study design, outcome measures, and enteral formula composition, limiting direct comparability across studies.

These differential macronutrient effects provide the physiological basis for why ED may reduce pancreatic stimulation. Clinical studies demonstrating the effects of ED on CCK release and pancreatic exocrine secretions are summarized in **Figure 2**. Vidon et al. demonstrated that nasojejunal infusion of an elemental formula resulted in substantially lower pancreatic lipase output compared with an isocaloric crushed food homogenate (Vidon et al., 1978). Notably, no fasting or placebo control was included in this study. In healthy volunteers, Shea et al. demonstrated that ED produced only a modest increase in circulating CCK relative to fasting, whereas levels rose substantially higher when the subjects were fed with a standard polymeric enteral formula or a high-fat meal, suggesting that elemental diets may weakly stimulate CCK release compared to more intact meals (Shea et al., 2003). In more controlled parallel-group physiology studies, Kaushik et al. showed that duodenal infusion of an ED modestly increased trypsin secretion above fasting levels, but to a significantly lesser extent than polymeric diets. ED did not increase lipase secretion above baseline, whereas polymeric diet resulted in a significant rise. Amylase secretion did not differ significantly between elemental, polymeric, and fasting conditions (Kaushik et al., 2005). Similar findings were reported by O’Keefe et al., who used a comparable experimental design. In that study, duodenal ED feeding was associated with a modest increase in lipase secretion, again substantially less than with polymeric feeding, as there were significant increases in trypsin and amylase observed with polymeric diets (O’Keefe et al., 2003). Collectively, these studies consistently indicate that, while ED are not entirely non-stimulatory, they produce substantially lower pancreatic exocrine responses than polymeric diets. This supports the concept that nutrient form and composition can meaningfully modulate pancreatobiliary stimulation.

### 2.4 Environmental and other modulators

The regulation of pancreatic exocrine secretion involves a sophisticated interplay of neural, hormonal, and luminal factors that coordinate digestive enzyme and bicarbonate secretion in response to food intake. Among these, vagally mediated reflex pathways play an important role in adjusting pancreatic enzyme and bicarbonate secretion across the cephalic, gastric, and intestinal phases of digestion. During the cephalic phase, sensory cues such as the sight, smell, taste, mastication, and anticipation of food activate vagal pathways, leading to efferent vagal stimulation and acetylcholine (ACh) release at pancreatic acinar cells, which promotes enzyme secretion and enhances gastric secretion and gastrointestinal motility (Katschinski, 2000; Katschinski et al., 1992; Konturek et al., 2003; Pandol, 2011). The gastric phase adds further vagal stimulation through gastric distension and gastrin release from antral G cells (Konturek et al., 2003). In the intestinal phase, CCK acts both as a circulating hormone that directly affects pancreatic acinar cells and as a neural modulator. Released CCK activates vagal afferent neurons, which transmit signals to the dorsal vagal complex, and these inputs are integrated with sensory input and lead to the activation of vagal efferents that synapse within pancreatic ganglia. Through the release of neurotransmitters such as ACh, gastrin-releasing peptide (GRP), and vasoactive intestinal peptide (VIP), postganglionic neurons stimulate pancreatic parenchymal cell secretion (Hall and Hall, 2021; Pandol, 2011).

Luminal factors also modulate pancreatic secretions. The neurotransmitter serotonin (5-hydroxytryptamine, 5-HT) is released in response to luminal stimuli including acidity, hyperosmolarity, disaccharides, and mechanical distension. Activation of serotonin receptors on vagal afferent fibers indirectly modulates pancreatic enzyme secretion via vagovagal reflex pathways (Chey and Chang, 2001; Li et al., 2000). Experimental studies have further characterized the influence of luminal conditions. In dogs, Ragins et al. demonstrated that jejunal infusion of an elemental formula stimulated pancreatic secretion only at acidic pH (but not at neutral), highlighting the role of luminal pH (Ragins et al., 1973). In humans, Vidon et al., reported osmolality and infusion volume exert minimal influence when nutrient composition and delivery site are controlled (Vidon et al., 1978). In contrast, Schapiro reported infusion of hypertonic solutions into the upper small intestine of dogs inhibited secretin-stimulated pancreatic exocrine flow, suggesting hyperosmolality may exert inhibitory effects (Schapiro et al., 1967).

Pancreatic exocrine secretion is subject not only to stimulatory inputs, but also inhibitory feedback mechanisms. One proposed regulatory pathway is the “ileal brake,” a neurohormonal feedback system triggered by the delivery of nutrients – particularly fats – to the distal small intestine. Activation of this pathway slows GI motility and reduces proximal bowel digestive secretions, and its effects have been proposed to extend to attenuation of pancreatic exocrine secretion. Several gut-derived peptides have been implicated as potential mediators, including peptide YY (PYY), glucagon-like peptide-1 (GLP-1), and pancreatic polypeptide (PP) (Jung et al., 1987; Kaushik et al., 2005; Vu et al., 1999). Experimental studies support a potential inhibitory role of these mediators. Infusion of PYY in canine models decreases pancreatic blood flow as well as enzyme and bicarbonate secretion (Konturek et al., 1988). The direct effect of GLP-1 on pancreatic exocrine function remains less clear, with existing literature demonstrating inconsistent findings (Akalestou et al., 2016; Wewer Albrechtsen et al., 2016). Exogenous PP administration has been shown to attenuate pancreatic bicarbonate, enzyme, and bilirubin secretion (Adrian et al., 1979; Greenberg et al., 1978; Jung et al., 1987; Kayasseh et al., 1978). Separate from the ileal brake, somatostatin is a potent inhibitor of pancreatic exocrine function, and acts through multiple mechanisms, including direct inhibition of acinar cells, suppression of stimulatory hormone release, reduced secretin secretion, and decreased pancreatic flow rates, bicarbonate output, and protein secretion (Bali et al., 2006; Boden et al., 1975; Hildebrand et al., 1992).

In summary, pancreatic exocrine secretion reflects a dynamic balance between stimulatory signals, dominated by CCK, secretin, vagal cholinergic activity, and inhibitory mechanisms such as the ileal brake, PYY, and somatostatin. This network provides a physiological basis for how the location and composition of nutrient delivery can attenuate pancreatic stimulation and secretion, which we postulate is a component of pain in chronic pancreatitis.

## 3 Jejunal feeding as a therapeutic strategy

### 3.1 Current clinical evidence linking jejunal feeding and relief of chronic pancreatitis pain

The motivation and hypothesis of this review arise from observations in clinical practice, where select patients with CP and refractory abdominal pain have experienced meaningful improvement following targeted nutritional interventions. In our practice, individual patients plagued with symptoms despite conventional therapies were able to find significant and sustained improvement after initiation of jejunal feeding across multiple domains, including pain severity, nutritional status, physical and mental function, and overall quality of life. While singular cases cannot establish causality, they generate important questions and align with cohort studies and data involving nasojejunal feeding in acute pancreatitis suggesting that jejunal nutrition may be effective in reducing pain burden and analgesic requirements in selected CP populations (Moore et al., 2006; Skipworth et al., 2011; Stanga et al., 2005).

In a retrospective study by Stanga et al., 57 CP patients who were fed through jejunal access (53 via percutaneous endoscopic gastrojejunostomy (PEG-J) and 4 via direct percutaneous endoscopic jejunostomy (DPEJ)) were monitored for 6 months. The prevalence of persistent abdominal pain declined from 96% to 23% after 180 days (p<0.001), and the proportion of patients requiring pain medications decreased from 91% to 27% (p<0.001) (Stanga et al., 2005). Similarly, in a retrospective study by Skipworth et al., 58 patients were fed via nasojejunal route over a median of 47 days. 46/58 (79.3%) reported resolution of their abdominal pain and cessation of opioid analgesics over the study period (Skipworth et al., 2011). In another retrospective case series (reported in abstract form only), 25 CP patients were provided jejunal nutrition through various access routes (often with >1 route per patient: 13 via nasoenteric tubes, 9 via PEG-J, 14 via DPEJ). Prevalence of uncontrolled abdominal pain at the start of the study was reported in 20 (80%) of patients, and 9 (45%) achieved near complete or complete pain resolution (p=0.008) over a mean duration of 4.6 months of jejunal feeding (Moore et al., 2006).

Together, these clinical observations and retrospective studies suggest that jejunal feeding may confer meaningful analgesic benefits in selected patients with CP. However, the existing evidence remains limited, consisting of small, uncontrolled retrospective cohorts with heterogeneous patient populations, variable jejunal access routes, and differing formula compositions. Despite these limitations, the consistent signals of pain reduction and decreased opioid use across multiple reports provide a compelling physiological rationale and warrant further study. These findings raise important queries about how jejunal nutrition modulates pain in CP and by understanding more about underlying physiology, we can explore how to best optimize this potential therapeutic strategy.

### 3.2 Potential mechanisms of jejunal feeding on reducing pancreaticobiliary secretions, GI symptoms, and pain in chronic pancreatitis

**Figure 3:**
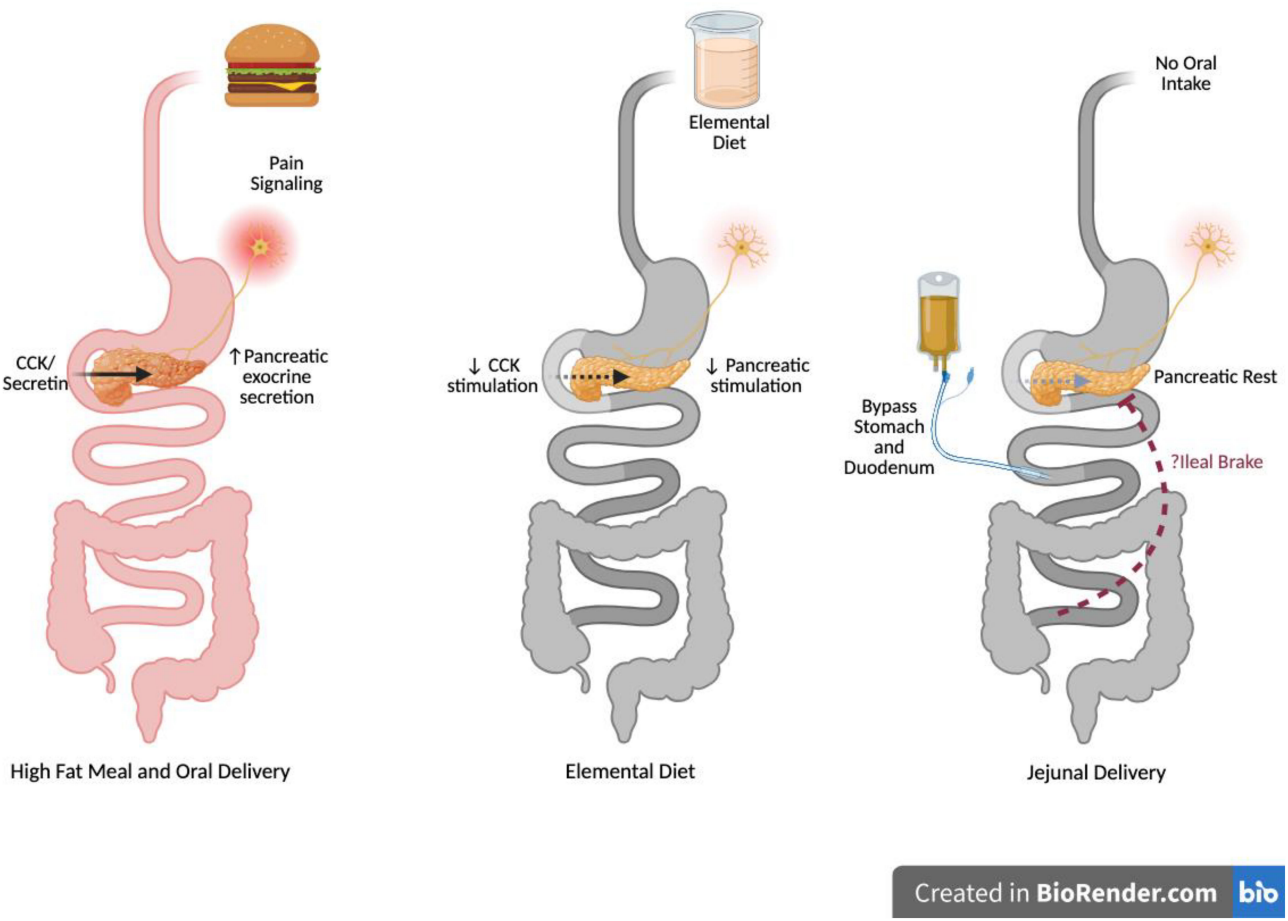
Proposed physiologic mechanism through which elemental feeding and jejunal feeding lead to decreased pain flares. The image illustrates the underlying hypothesis that regular, intact diets taken orally stimulate pancreatic exocrine secretion and thereby induce pain signaling. Feeding with elemental diets composed of free amino acids, MCTs, and simple carbohydrates allows rapid absorption with minimal CCK stimulation. Thus, this may result in less pancreatic enzyme secretion into the injured pancreatic duct and pancreas, potentially decreasing CP pain exacerbation. Jejunal delivery bypasses the stomach and duodenum, resulting in minimal pancreatic activation (namely “pancreatic rest”) and potentially engaging in the ileal brake mechanism to collectively decrease pancreatic pain exacerbation. Created with BioRender.com and modified by the authors.

Jejunal feeding delivers nutrients past the mouth, stomach, and duodenum, thereby potentially reducing pancreatic stimulation brought on during the cephalic, gastric, and proximal intestinal phases of a meal while maintaining nutritional support. Although prior studies have used variable jejunal infusion sites, clinical data reviewed in Section III.B consistently support that more distal intestinal feeding is associated with reduced pancreatic activation and exocrine secretions, in some reports approaching levels seen in parenteral nutrition (Kaushik et al., 2005; Vu et al., 1999). This concept of “pancreatic rest” has been proposed as one mechanism that may contribute to the relief in CP-associated pain and improvement in GI symptoms. Across the studies by Vu et al. and Kaushik et al., nutrient delivery to the duodenum or proximal jejunum in healthy individuals was associated with significant increases in pancreatic enzyme secretion, whereas more distal feeding >40 cm from the LoT had significantly less output (Kaushik et al., 2005; Vu et al., 1999). In those with CP, Stanga et al. observed significant reductions in plasma amylase and lipase levels over six months of jejunal feeding. When considered alongside the marked improvement in pain symptoms and decreased narcotic use observed in the same study, as well as similar clinical benefits reported by Skipworth et al. and Moore et al., these findings support the hypothesis that physiologic processes associated with increased pancreatic enzyme secretion may contribute to pain generation in some CP phenotypes (Moore et al., 2006; Skipworth et al., 2011; Stanga et al., 2005). By reducing pancreatic exocrine stimulation, jejunal feeding may help interrupt a self-reinforcing cycle of pancreatic injury and pain amplification, thereby attenuating inflammatory signaling, peripheral nociception activation, and ductal/parenchymal edema, and may ultimately help decrease the pain and improve quality of life that CP patients experience (**Figure 3**).

One can also consider that in the setting of impaired ductal anatomy or outflow obstruction, decreased enzyme and bicarbonate secretion may also reduce pancreatic ductal hypertension, another contributor implicated in pain generation in CP (A. Singh et al., 2023). Additionally, many patients with CP have coexisting GI disorders, such as gastroparesis, which can independently contribute to nausea and abdominal discomfort (Chowdhury et al., 2003). Direct jejunal feeding bypasses the stomach and can circumvent gastric dysmotility, potentially alleviating these symptoms, while concomitant reduction in opioid use observed in the clinical studies is also likely to further improve GI symptoms beyond pain.

In addition to reductions in pancreatic stimulation through proximal gut bypass and decreased CCK-mediated pathways, activation of distal intestinal feedback pathways may be another potential – though less well-characterized – physiological mechanism contributing to pancreatic suppression during jejunal feeding. As discussed previously, nutrient delivery to the distal small intestine can activate the “ileal brake”, a feedback system that slows proximal GI motility and may influence pancreatic exocrine function. Human studies demonstrate that jejunal nutrient delivery can significantly increase circulating PYY and GLP-1, and animal models show PYY infusion inhibits pancreatic exocrine secretion (Hu et al., 2017; Jin et al., 1993; Kaushik et al., 2005; Robinson et al., 1996; Vu et al., 1999). These findings raise the possibility that distal nutrient exposure during jejunal feeding could contribute to attenuation of pancreatic activity. However, the other aspects of this mechanism remain unclear. The role of GLP-1 signaling in pancreatic exocrine regulation is particularly controversial, with preclinical, clinical, and epidemiological data yielding mixed findings regarding the relationship between GLP-1 signaling, pancreatic exocrine function, and pancreatitis risk (Akalestou et al., 2016; Alenzi et al., 2024; Ayoub et al., 2025; FDA, 2025; Satoh et al., 2019; S. Singh et al., 2013; Vallejo et al., 2024; Wewer Albrechtsen et al., 2016; Xie et al., 2025). Additionally, while exogenous PP administration appears to attenuate pancreatic exocrine function, human studies have not shown any significant rise in circulating PP with intrajejunal feeding (Adrian et al., 1979; Greenberg et al., 1978; Jung et al., 1987; Kaushik et al., 2005; Kayasseh et al., 1978; Vu et al., 1999). Consequently, while ileal brake activation may complement the reduction of pancreatic secretion associated with jejunal feeding, its role remains incompletely characterized.

Collectively, available pathophysiological data supports mid-to-distal jejunal feeding as an effective strategy to reduce pancreatic stimulation, which may decrease exocrine enzyme secretion into an injured pancreas – a potential contributor to pain in selected patients with CP. Jejunal feeding may also reduce pancreatic ductal hypertension through decreased secretory volume and possibly engage the ileal brake mechanism, thereby reinforcing pancreatic rest and contributing to the symptom improvement observed in multiple clinical studies.

### 3.3 Role of elemental diet in nutrition management and pain reduction in chronic pancreatitis

Beyond the site of nutrient delivery, formula composition may influence pain and GI symptoms in CP. Compared to standard feeds, elemental diets are associated with reduced CCK release and pancreatic exocrine secretion. Shea et al. associated reduced pancreatic stimulation in healthy individuals with potentially clinically meaningful benefits. In a separate arm of the same report, a cohort of eight CP patients with pain refractory to multiple other therapies experienced a 68.5% reduction in median pain scores following oral ED supplementation (p=0.011) (Shea et al., 2003). In a Japanese prospective observational study of 596 patients with CP, daily supplementation with approximately 600 kcal of an elemental diet was associated with significant reduction of abdominal pain and discomfort, with visual analog scale (VAS) scores decreasing from 52.9 to 20.0 mm at 12 weeks (p<0.001), alongside improvement in nutritional indices; patients with baseline BMI<25 experienced significant increases in BMI (Kataoka et al., 2014). Consistent results were observed in another prospective cohort study of 17 patients with CP and chronic pain, where ED supplementation over 8 weeks led to pain improvement in 88% of the cohort and complete pain resolution in 58.8% (Ikeura et al., 2014).

Elemental diets may confer benefits beyond pain control. Their fully hydrolyzed composition has been shown to improve gut barrier function, modulate intestinal microbiome, and reduce intestinal inflammatory signaling (Nasser et al., 2024). Lacking intact proteins, ED are essentially allergen-free and may attenuate immune-mediated gut inflammation (Nasser et al., 2024). Preclinical studies support an additional microbiota-mediated mechanism to help gut integrity: rats fed elemental diets exhibited reduced mucolytic bacterial populations, preservation of the intestinal mucosal layer, and decreased microbial invasion (B. Zhang et al., 2022). ED may also reduce symptom triggers by limiting fermentable substrates, thereby decreasing luminal distension and visceral pain - an effect particularly relevant in CP, where comorbid irritable bowel syndrome and intestinal microbial overgrowth are common (Lee et al., 2019; Leeds et al., 2010). In a human SIBO study, two weeks of exclusive elemental diet therapy normalized the breath test in 73% of participants, with 83% reporting adequate symptom relief (Rezaie et al., 2025). Additionally, ED typically excludes food additives and emulsifiers, which have been implicated in impaired gut integrity, inflammation, and metabolic or allergic disorders (Naimi et al., 2021; Seto et al., 2025; Urrutia-Pereira et al., 2025).

Together, these findings situate ED as a nutritional strategy capable of modulating pancreatic stimulation through nutrient composition. Like jejunal feeding, the same physiological principles of relieving pain in CP through decreased pancreatic secretions may be leveraged with oral elemental diets in patients who wish to avoid instrumentation or in whom tube feeding is not feasible. Historically, the use of oral elemental diets has been limited by poor palatability, despite the availability of several commercial products. A newer formulation (mBIOTA Elemental) has been developed with the aim of improving taste and tolerability. Anecdotally, this new formulation is more tolerable and palatable than previous ones on the market, which may lead to enhanced adherence; however, its feasibility and tolerability in CP patients remains to be established (Rezaie et al., 2025).

When combined with distal jejunal feeding, elemental diets may offer synergistic benefits in CP patients by bypassing the proximal small bowel to limit pancreatic stimulation while delivering highly absorbable nutrients to the distal small bowel. This positions jejunal elemental feeding as a potential therapeutic strategy for selected patients with CP, aiming to achieve both pain control and nutritional rehabilitation, with additional potential benefits for gut integrity and alleviation of other concomitant GI symptoms.

### 3.4 Extended benefits of jejunal feeding beyond symptom control

If the therapeutic goal is to minimize pancreatic exocrine secretion, one may ask why not rely solely on parenteral nutrition (PN) and bypass the GI tract entirely? In chronic pancreatitis, PN is generally only done in situations in which enteral nutrition (EN) is not feasible (e.g. gastric outlet obstruction requiring decompression, complex fistulizing disease, or intolerance or inability to place/maintain a jejunal feeding tube). The preference to avoid PN when possible reflects the broader medical community’s perspective, as EN is known to better preserve mucosal integrity and immune function, while avoiding the risks that come with PN, including catheter-associated infections, thrombosis, and metabolic complications (Arvanitakis et al., 2024; G. Zhang et al., 2018). Among nutritional strategies aimed at minimizing pancreatic stimulation, distal (jejunal) feeding may offer a practical compromise by supporting enteral nutrition while still pursuing “pancreatic rest” (Baik et al., 2024; Berlana, 2022; Wan et al., 2015).

Jejunal feeding may also improve nutritional status and overall clinical outcomes in patients with CP. In a large cohort study, Ridtitid et al. followed 102 patients who underwent PEG-J placement, and found that, among the 53 patients with CP, jejunal feeding was associated with increases in BMI and fewer hospitalizations (Ridtitid et al., 2017). In patients with CP, Hamvas et al. reported that those receiving nasojejunal enteral feeding (n=12) experienced faster clinical recovery and required fewer interventions than those managed with parenteral nutrition (n=7) (Hamvas et al., 2001). Consistent with these findings, the retrospective studies discussed in Section IV.A demonstrated improvements not only in pain-related outcomes, but also with nutritional parameters. For example, Stanga et al. reported a significant increase in mean body weight from 64.8 kg to 69.1 kg at six months (p < 0.001), alongside a rise in serum albumin from 2.6 g/dL to 3.4 g/dL over six months (p < 0.001) (Stanga et al., 2005). Similarly, Skipworth et al. observed modest weight gain over a median of 47 days (60.0 kg to 61.5 kg, p = 0.454) accompanied by significant improvements in laboratory markers, including increased albumin (34.5 g/L to 38.7 g/L, p = 0.002), higher sodium, creatinine, and hemoglobin levels, as well as a reduction in C-reactive protein (CRP) (Skipworth et al., 2011). While these observational data are subject to potential confounding factors (e.g., concurrent dietician support, optimization of PERT, or regression to the mean), and weight and serological markers remain imperfect proxies for nutritional status – since, for example, albumin can reflect inflammation or clinical acuity rather than nutrition alone – the consistent trends across multiple studies, even over relatively short follow-up periods, support the notion that jejunal feeding may meaningfully improve nutritional status and clinical outcomes for selected patients with CP.

### 3.5 Limitations and technical challenges

Despite compelling supporting data for the benefits of jejunal feeding with ED, it is not without its limitations. One concern is the absorptive capacity in the distal small intestine. Although enteral feeds contain daily vitamins and mineral requirements, certain micronutrients (particularly iron and folate) are absorbed most efficiently in the duodenum and proximal small bowel (Malik et al., 2025). Patients who undergo Roux-en-Y gastric bypass frequently develop deficiencies in iron, vitamin B12, folate, and thiamine, in part, due to the bypass of absorption in the duodenum and proximal small bowel (Nuzzo et al., 2021; Saltzman and Karl, 2013). However, even though direct studies evaluating micro- and macronutrient absorption during long-term jejunal feeding are limited, available clinical experience and small series suggest that patients generally tolerate it well and can maintain adequate nutritional status. Prolonged exclusive use of MCT-based formulas also necessitates essential fatty acid supplementation, particularly linoleic acid (Jadhav and Annapure, 2023). Intolerance to jejunal or ED feeding – manifesting as abdominal pain, cramping, diarrhea, or nausea – can limit therapy in a subset of patients. In Stanga et al., eight patients (14%) receiving jejunal feeding were intolerant to standard tube feeds; half improved with transition to ED, while the rest (7%) continued to have intolerable symptoms and required device removal (Stanga et al., 2005). Skipworth et al. reported “poor” tolerance in four (7%) patients fed through nasojejunal tubes, and Kataoka et al. observed adverse reactions in 31 patients (5.21%) with oral ED, most commonly diarrhea, hyperglycemia, and abdominal bloating (Kataoka et al., 2014; Skipworth et al., 2011). Altogether, the need to assess nutritional status and tolerance underscores the importance of close monitoring, individualized nutritional plans and supplementation, ideally under the supervision of an experienced dietitian.

Placement of a permanent or semi-permanent tube is itself a logistical barrier. The PEG-J is the most commonly used since it is relatively easier to place, but the jejunal extension tube, given its smaller caliber, is prone to clogging, kinking, or migration and typically requires replacement every few weeks to months (often ~4–12 weeks). DPEJ or surgically placed jejunostomy tubes tend to be more durable with fewer tube-related complications, but their placement needs more specialized expertise and equipment, limiting availability. As such, the ultimate decision should be individualized, taking into account patient-specific factors and institutional resources (Itkin et al., 2011; Krafft et al., 2025). Successful insertion rates of jejunal tubes are high across the available studies; however, with any indwelling device, jejunal enteral tubes are inherently associated with risk. They are usually minor, with infrequent serious adverse events. In Ridtitid et al., PEG-J placement had a 97% technical success rate, and no documented major complications (Ridtitid et al., 2017). Stanga et al. documented one wound infection (which was managed conservatively) and a single colonic mesenteric injury requiring emergent laparotomy (1.8%), which the patient was able to recover from (Stanga et al., 2005).

An additional consideration of jejunal feeding with an elemental diet is the training and time commitment placed on the patient themselves, which can limit spontaneity and pose challenges for travel or social events; however, portable feeding systems are becoming increasingly user-friendly and adaptable to daily life (Reddick et al., 2023; White and King, 2014). Despite practical burdens, patients may experience substantial pain relief and improved symptom control, which can translate to gains in their quality of life and greater functional independence. Implementation of these systems would also greatly benefit from ongoing support from a multidisciplinary team (including nurses, technicians, pharmacists, dieticians, and physicians) to further optimize outcomes. With thoughtful patient selection and alignment of individualized goals of care, jejunal feeding with ED has potential to be a valuable therapeutic option for pain-predominant chronic pancreatitis, provided that its benefits are balanced against nutritional, technical, and patient-centered considerations.

## 4 Conclusion

Chronic pancreatitis remains a therapeutic challenge, where intractable pain and nutritional compromise often persist despite multimodal intervention (Goulden, 2013; Hines and Pandol, 2024; Poulsen et al., 2013). Although a direct causal link between pancreatic exocrine secretion and pain in chronic pancreatitis has not been formally established, clinical observations suggest that attenuating pancreatic stimulation provides a “rest” state that can break the cycle of postprandial distress and decrease overall pain. Current clinical evidence indicates that mid-to-distal jejunal feeding is associated with reductions in pancreatic secretions, abdominal pain, and opioid requirements while stabilizing or even improving nutritional markers (Cassim and Allardyce, 1974; Hamvas et al., 2001; Kaushik et al., 2005; Moore et al., 2006; O’Keefe et al., 2003; Skipworth et al., 2011; Stanga et al., 2005; Vu et al., 1999). Elemental diets may confer similar therapeutic benefits through attenuation of pancreatic stimulation, and may be used independently or in combination with jejunal delivery (Kataoka et al., 2014; Kaushik et al., 2005; Shea et al., 2003; Symersky et al., 2002; Vidon et al., 1978; Watanabe et al., 1986). This approach offers the physiological benefits of “gut rest” typically achieved by parenteral nutrition, but without the significant infectious and metabolic risks of an intravenous route (Arvanitakis et al., 2024).

Despite these encouraging signals, the current evidence base is limited by the nature of existing studies. Physiology and mechanistic inferences are largely extrapolated from experiments in healthy volunteers or animal models, as direct measurements of pancreatic exocrine secretion in symptomatic CP patients – especially in conditions hypothesized to provoke pain – poses ethical challenges. The absence of high-quality prospective, randomized controlled trials represents a significant gap in the field. Future study designs should focus on patient phenotyping, standardizing feeding routes and formulation composition, and standardizing outcomes. Comparator trials evaluating different commercially available enteral formulations would be helpful to assess their effects on pancreatic stimulation and pain outcomes. Randomized crossover designs, including N-of-1 trials, may be particularly well suited to CP given the inherent symptom variability of CP. Such approaches could help isolate the benefits of nutritional interventions and filter out the noise of concurrent analgesic adjustments and procedural therapies, psychosocial factors, and comorbid conditions. Incorporation of tools such as Pancreatic Quantitative Sensory Testing (PQST) may help identify patients most likely to respond to strategies aimed at pancreatic secretory suppression. In parallel, emerging *in silico* New Approach Methodologies (NAMs), including systems biology modeling and computational simulation, can offer the potential to interrogate complex neurohormonal networks involved in pancreatic secretion (e.g. CCK, secretin, PYY, somatostatin, and PP) and downstream pain modulation to generate hypotheses and inform rational comparative trials (Charest et al., 2025; Marques et al., 2024).

In summary, jejunal elemental feeding represents more than mere caloric support; it is a biologically plausible therapeutic adjunct that may mitigate pain, dysmotility, and malnutrition in select patients with chronic pancreatitis. Translating physiologic insight into validated, individualized clinical care will require prospective randomized trials and mechanistically informed strategies. Such efforts have the potential to expand the therapeutic landscape for individuals with pain-predominant chronic pancreatitis.

## References

[B1] Adrian T. E. Besterman H. S. Mallinson C. N. Greenberg G. R. Bloom S. R. (1979). Inhibition of secretin stimulated pancreatic secretion by pancreatic polypeptide. Gut 20, 37–40. doi: 10.1136/gut.20.1.37 761835 PMC1418966

[B2] Ahmed Ali U. Pahlplatz J. M. Nealon W. H. Van Goor H. Gooszen H. G. Boermeester M. A. (2015). Endoscopic or surgical intervention for painful obstructive chronic pancreatitis. Cochrane Database Systematic Rev. 2015. doi: 10.1002/14651858.CD007884.pub3 25790326 PMC10710281

[B3] Akalestou E. Christakis I. Solomou A. M. Minnion J. S. Rutter G. A. Bloom S. R. (2016). Proglucagon-derived peptides do not significantly affect acute exocrine pancreas in rats. Pancreas 45, 967–973. doi: 10.1097/MPA.0000000000000585 26731187 PMC4820085

[B4] Alenzi K. A. Alsuhaibani D. Batarfi B. Alshammari T. M. (2024). Pancreatitis with use of new diabetic medications: A real-world data study using the post-marketing FDA adverse event reporting system (FAERS) database. Front. Pharmacol. 15, 1364110. doi: 10.3389/fphar.2024.1364110 38860168 PMC11163090

[B5] Arvanitakis M. Ockenga J. Bezmarevic M. Gianotti L. Krznarić ŽCheckt. a. e. Lobo D. N. . (2024). ESPEN practical guideline on clinical nutrition in acute and chronic pancreatitis. Clin. Nutr. 43, 395–412. doi: 10.1016/j.clnu.2023.12.019 38169174

[B6] Ayoub M. Chela H. Amin N. Hunter R. Anwar J. Tahan V. . (2025). Pancreatitis risk associated with GLP-1 receptor agonists, considered as a single class, in a comorbidity-free subgroup of type 2 diabetes patients in the United States: A propensity score-matched analysis. J. Clin. Med. 14, 944. doi: 10.3390/jcm14030944 39941615 PMC11818918

[B7] Baik S. M. Kim M. Lee J. G. (2024). Comparison of early enteral nutrition versus early parenteral nutrition in critically ill patients: A systematic review and meta-analysis. Nutrients 17, 10. doi: 10.3390/nu17010010 39796444 PMC11723109

[B8] Bali M. A. Golstein P. Devière J. Chatterjee N. Matos C. (2006). Evaluation of somatostatin inhibitory effect on pancreatic exocrine function using secretin-enhanced dynamic magnetic resonance cholangiopancreatography: A crossover, randomized, double blind, placebo-controlled study. Pancreas 32, 346–350. doi: 10.1097/01.mpa.0000220858.93496.a8 16670616

[B9] Bampton T. J. Chen J. W. Brown A. Barnett M. I. Coates P. T. Palmer L. J. (2025). Epidemiology and burden of adult chronic pancreatitis in South Australia: A 20-year data linkage study. BMJ Open 15, e089297. doi: 10.1136/bmjopen-2024-089297 40050052 PMC11887304

[B10] Berlana D. (2022). Parenteral nutrition overview. Nutrients 14, 4480. doi: 10.3390/nu14214480 36364743 PMC9659055

[B11] Bertin C. Pelletier A.-L. Vullierme M. P. Bienvenu T. Rebours V. Hentic O. . (2012). Pancreas divisum is not a cause of pancreatitis by itself but acts as a partner of genetic mutations. Am. J. Gastroenterol. 107, 311–317. doi: 10.1038/ajg.2011.424 22158025

[B12] Beyer G. Habtezion A. Werner J. Lerch M. M. Mayerle J. (2020). Chronic pancreatitis. Lancet 396, 499–512. doi: 10.1016/S0140-6736(20)31318-0 32798493

[B13] Boden G. Sivitz M. C. Owen O. E. Essa-Koumar N. Landor J. H. (1975). Somatostatin suppresses secretin and pancreatic exocrine secretion. Science 190, 163–165. doi: 10.1126/science.1166308 1166308

[B14] Bondesen S. Christensen H. Lindorff-Larsen K. Schaffalitzky de Muckadell O. B. (1985). Plasma secretin in response to pure bile salts and endogenous bile in man. Digestive Dis. Sci. 30, 440–444. doi: 10.1007/BF01318176 3987477

[B15] Cai Q.-Y. Tan K. Zhang X.-L. Han X. Pan J.-P. Huang Z.-Y. . (2023). Incidence, prevalence, and comorbidities of chronic pancreatitis: A 7-year population-based study. World J. Gastroenterol. 29, 4671–4684. doi: 10.3748/wjg.v29.i30.4671 37662860 PMC10472896

[B16] Cassim M. M. Allardyce D. B. (1974). Pancreatic secretion in response to jejunal feeding of elemental diet. Ann. Surg. 180, 228–231. doi: 10.1097/00000658-197408000-00017 4210477 PMC1343643

[B17] Chan L. K. M. Rao T. Masangcay P. Kuo S. C. L. Wan T.-T. (2025). A systematic review and meta-analysis of the efficacy of endoscopic ultrasound guided celiac plexus blocks for chronic pancreatitis pain. J. Pain Palliative Care Pharmacotherapy 39, 254–265. doi: 10.1080/15360288.2025.2479481 40168184

[B18] Charest N. Sinclair G. Eytcheson S. A. Chang D. T. Martin T. M. Lowe C. N. . (2025). Combined *in vitro* and in silico workflow to deliver robust, transparent, and contextually rigorous models of bioactivity. J. Chem. Inf. Model. 65, 4426–4441. doi: 10.1021/acs.jcim.5c00713 40273369 PMC13276861

[B19] Chey W. Y. Chang T. (2001). Neural hormonal regulation of exocrine pancreatic secretion. Pancreatology 1, 320–335. doi: 10.1159/000055831 12120211

[B20] Chowdhury R. S. Forsmark C. E. Davis R. H. Toskes P. P. Verne G. N. (2003). Prevalence of gastroparesis in patients with small duct chronic pancreatitis. Pancreas 26, 235–238. doi: 10.1097/00006676-200304000-00005 12657948

[B21] Dalley A. F. Agur A. M. R. Moore K. L. (2023). “ Clinically oriented anatomy,” in Moore’s Clinically Oriented Anatomy, 9th edition (Philadelphia, PA: Wolters Kluwer), 410–558.

[B22] Domínguez-Muñoz J. E. Phillips M. (2018). Nutritional therapy in chronic pancreatitis. Gastroenterol. Clinics North. America 47, 95–106. doi: 10.1016/j.gtc.2017.09.004 29413021

[B23] Drewes A. M. Bouwense S. A. W. Campbell C. M. Ceyhan G. O. Delhaye M. Demir I. E. . (2017). Guidelines for the understanding and management of pain in chronic pancreatitis. Pancreatology 17, 720–731. doi: 10.1016/j.pan.2017.07.006 28734722

[B24] Dyck W. P. (1971). Influence of intrajejunal glucose on pancreatic exocrine function in man. Gastroenterology 60, 864–869. doi: 10.1016/S0016-5085(71)80086-0 5581330

[B25] FDA (2025). “ WARNING LETTER novo nordisk inc. MARCS-CMS 716495,” in Center for Drug Evaluation and Research (CDER). Available online at: https://www.fda.gov/inspections-compliance-enforcement-and-criminal-investigations/warning-letters/novo-nordisk-inc-716495-09092025 (Accessed September 9, 2025).

[B26] Gardner T. B. Adler D. G. Forsmark C. E. Sauer B. G. Taylor J. R. Whitcomb D. C. (2020). ACG clinical guideline: Chronic pancreatitis. Am. J. Gastroenterol. 115, 322–339. doi: 10.14309/ajg.0000000000000535 32022720

[B27] Go V. L. W. Gukovskaya A. Pandol S. J. (2005). Alcohol and pancreatic cancer. Alcohol. 35, 205–211. doi: 10.1016/j.alcohol.2005.03.010 16054982

[B28] Goodarzi M. O. Petrov M. S. Andersen D. K. Hart P. A. (2021). Diabetes in chronic pancreatitis: Risk factors and natural history. Curr. Opin. Gastroenterol. 37, 526–531. doi: 10.1097/MOG.0000000000000756 34074860 PMC8364494

[B29] Goulden M. R. (2013). The pain of chronic pancreatitis: A persistent clinical challenge. Br. J. Pain 7, 8–22. doi: 10.1177/2049463713479230 26516493 PMC4590150

[B30] Greenberg G. R. McCloy R. F. Adrian T. E. Chadwick V. S. Baron J. H. Bloom S. R. (1978). Inhibition of pancreas and gallbladder by pancreatic polypeptide. Lancet 2, 1280–1282. doi: 10.1016/s0140-6736(78)92042-1 82783

[B31] Hall J. Hall M. (2021). “ Guyton and hall textbook of medical physiology,” in Textbook of Medical Physiology, 14th edition (Philadelphia, PA: Elsevier), 744–753, 764–788.

[B32] Hamvas J. Schwab R. Pap A. (2001). Jejunal feeding in chronic pancreatitis with severe necrosis. JOP: J. Pancreas 2, 112–116. doi: 10.1016/S0899-9007(00)00508-6 11870333

[B33] Hanssen L. E. (1980). Pure synthetic bile salts release immunoreactive secretin in man. Scandinavian J. Gastroenterol. 15, 461–463. doi: 10.3109/00365528009181501 7433910

[B34] Hart P. A. Bellin M. D. Andersen D. K. Bradley D. Cruz-Monserrate Z. Forsmark C. E. . (2016). Type 3c (pancreatogenic) diabetes mellitus secondary to chronic pancreatitis and pancreatic cancer. Lancet Gastroenterol. Hepatol. 1, 226–237. doi: 10.1016/S2468-1253(16)30106-6 28404095 PMC5495015

[B35] Hildebrand P. Ensinck J. W. Gyr K. Mossi S. Leuppi J. Eggenberger C. . (1992). Evidence for hormonal inhibition of exocrine pancreatic function by somatostatin 28 in humans. Gastroenterology 103, 240–247. doi: 10.1016/0016-5085(92)91119-o 1351858

[B36] Hines O. J. Pandol S. J. (2024). Management of chronic pancreatitis. BMJ, e070920. doi: 10.1136/bmj-2023-070920 38408777

[B37] Hirota M. Shimosegawa T. Masamune A. Kikuta K. Kume K. Hamada S. . (2012). The sixth nationwide epidemiological survey of chronic pancreatitis in Japan. Pancreatology 12, 79–84. doi: 10.1016/j.pan.2012.02.005 22487515

[B38] Hu C. Wen L. Deng L. Zhang C. Lugea A. Su H.-Y. . (2017). The differential role of human cationic trypsinogen (PRSS1) p.R122H mutation in hereditary and nonhereditary chronic pancreatitis: A systematic review and meta-analysis. Gastroenterol. Res. Pract. 2017, 9505460. doi: 10.1155/2017/9505460 29118810 PMC5651130

[B39] Ikeura T. Takaoka M. Uchida K. Miyoshi H. Okazaki K. (2014). Beneficial effect of low-fat elemental diet therapy on pain in chronic pancreatitis. Int. J. Chronic Dis. 2014, 1–5. doi: 10.1155/2014/862091 26464866 PMC4590936

[B40] Issa Y. Kempeneers M. A. Bruno M. J. Fockens P. Poley J.-W. Ahmed Ali U. . (2020). Effect of early surgery vs endoscopy-first approach on pain in patients with chronic pancreatitis: The ESCAPE randomized clinical trial. JAMA 323, 237–247. doi: 10.1001/jama.2019.20967 31961419 PMC6990680

[B41] Itkin M. DeLegge M. H. Fang J. C. McClave S. A. Kundu S. Janne d’Othee B. . (2011). Multidisciplinary practical guidelines for gastrointestinal access for enteral nutrition and decompression from the Society of Interventional Radiology and American Gastroenterological Association (AGA) Institute, with endorsement by Canadian Interventional Radiological Association (CIRA) and Cardiovascular and Interventional Radiological Society of Europe (CIRSE). J. Vasc. Interventional Radiol. 22, 1089–1106. doi: 10.1016/j.jvir.2011.04.006 21782465

[B42] Jadhav H. B. Annapure U. S. (2023). Triglycerides of medium-chain fatty acids: A concise review. J. Food Sci. Technol. 60, 2143–2152. doi: 10.1007/s13197-022-05499-w 35761969 PMC9217113

[B43] Jin H. Cai L. Lee K. Chang T. M. Li P. Wagner D. . (1993). A physiological role of peptide YY on exocrine pancreatic secretion in rats. Gastroenterology 105, 208–215. doi: 10.1016/0016-5085(93)90028-b 8514036

[B44] Jung G. Louie D. S. Owyang C. (1987). Pancreatic polypeptide inhibits pancreatic enzyme secretion via a cholinergic pathway. Am. J. Physiol. 253, G706–G710. doi: 10.1152/ajpgi.1987.253.5.G706 2446510

[B45] Kataoka K. Sakagami J. Hirota M. Masamune A. Shimosegawa T. (2014). Effects of oral ingestion of the elemental diet in patients with painful chronic pancreatitis in the real-life setting in Japan. Pancreas 43, 451–457. doi: 10.1097/MPA.0000000000000038 24622078

[B46] Katschinski M. (2000). Nutritional implications of cephalic phase gastrointestinal responses. Appetite 34, 189–196. doi: 10.1006/appe.1999.0280 10744909

[B47] Katschinski M. Dahmen G. Reinshagen M. Beglinger C. Koop H. Nustede R. . (1992). Cephalic stimulation of gastrointestinal secretory and motor responses in humans. Gastroenterology 103, 383–391. doi: 10.1016/0016-5085(92)90825-j 1634057

[B48] Kaushik N. Pietraszewski M. Holst J. J. O’Keefe S. J. D. (2005). Enteral feeding without pancreatic stimulation. Pancreas 31, 353–359. doi: 10.1097/01.mpa.0000183374.11919.e5 16258370

[B49] Kayasseh L. Haecki W. H. Gyr K. Stalder G. A. Rittmann W. W. Halter F. . (1978). The endogenous release of pancreatic polypeptide by acid and meal in dogs. Effect of somatostatin. Scandinavian J. Gastroenterol. 13, 385–391. doi: 10.3109/00365527809181911 675146

[B50] Kempeneers M. A. Issa Y. Verdonk R. C. Bruno M. Fockens P. Van Goor H. . (2021). Pain patterns in chronic pancreatitis: A nationwide longitudinal cohort study. Gut 70, 1724–1733. doi: 10.1136/gutjnl-2020-322117 33158979

[B51] Khurmatullina A. R. Andreev D. N. Maev I. V. Kucheryavyy Y. A. Beliy P. A. Dzhafarova A. R. . (2025). Prevalence and risk of sarcopenia in patients with chronic pancreatitis: Systematic review and meta-analysis. Nutrients 17, 870. doi: 10.3390/nu17050870 40077740 PMC11902046

[B52] Konturek S. J. Bilski J. Pawlik W. Tasler J. Domschke W. (1988). Adrenergic pathway in the inhibition of pancreatic secretion by peptide YY in dogs. Gastroenterology 94, 266–273. doi: 10.1016/0016-5085(88)90412-x 3335306

[B53] Konturek S. J. Pepera J. Zabielski K. Konturek P. C. Pawlik T. Szlachcic A. . (2003). Brain-gut axis in pancreatic secretion and appetite control. J. Physiol. Pharmacol: An. Off. J. Polish Physiol. Soc. 54, 293–317. 14566070

[B54] Krafft M. R. Maan S. Scott A. Shepherd K. Karna R. Clemetson E. . (2025). Percutaneous endoscopic gastrostomy with jejunal extension versus direct percutaneous endoscopic jejunostomy for post-pyloric feeding: A dual-center retrospective study. Digestive Dis. Sci. 70, 4190–4206. doi: 10.1007/s10620-025-09198-2 40736945

[B55] Lawrence W. Khentigan A. Hudock J. Vanamee P. (1961). The effect of intraduodenal administration of hypertonic glucose solution on external pancreatic secretion. Surgery 49, 666–675. 13759704

[B56] Lee A. A. Baker J. R. Wamsteker E. J. Saad R. DiMagno M. J. (2019). Small intestinal bacterial overgrowth is common in chronic pancreatitis and associates with diabetes, chronic pancreatitis severity, low zinc levels, and opiate use. Am. J. Gastroenterol. 114, 1163–1171. doi: 10.14309/ajg.0000000000000200 31008737 PMC6610753

[B57] Leeds J. S. Hopper A. D. Sidhu R. Simmonette A. Azadbakht N. Hoggard N. . (2010). Some patients with irritable bowel syndrome may have exocrine pancreatic insufficiency. Clin. Gastroenterol. Hepatol: Off. Clin. Pract. J. Am. Gastroenterol Assoc. 8, 433–438. doi: 10.1016/j.cgh.2009.09.032 19835990

[B58] Li Voti R. Macaluso F. S. Banci E. Campanozzi A. D’Arcangelo G. De Blasi A. . (2025). The role of nutritional therapy in the treatment of adults with Crohn’s disease: A review. Nutrients 17, 3186. doi: 10.3390/nu17203186 41156439 PMC12567425

[B59] Li Y. Hao Y. Zhu J. Owyang C. (2000). Serotonin released from intestinal enterochromaffin cells mediates luminal non-cholecystokinin-stimulated pancreatic secretion in rats. Gastroenterology 118, 1197–1207. doi: 10.1016/s0016-5085(00)70373-8 10833495

[B60] Lin Z. Pandol S. Apte M. Jiang Y. (2025). Navigating chronic pancreatitis pain: A pathophysiological and therapeutic overview. Front. Physiol. 16, 1622845. doi: 10.3389/fphys.2025.1622845 40708786 PMC12287759

[B61] Lu V. B. Gribble F. M. Reimann F. (2021). Nutrient-induced cellular mechanisms of gut hormone secretion. Nutrients 13, 883. doi: 10.3390/nu13030883 33803183 PMC8000029

[B62] Machicado J. D. Dudekula A. Tang G. Xu H. Wu B. U. Forsmark C. E. . (2019). Period prevalence of chronic pancreatitis diagnosis from 2001–2013 in the commercially insured population of the United States. Pancreatology 19, 813–818. doi: 10.1016/j.pan.2019.07.003 31350077 PMC6756969

[B63] Machicado J. D. Tanner S. Adoor D. Chalhoub J. M. Plann-Curley B. Lee U. J. . (2025). Endoscopic ultrasound-guided celiac plexus block for painful chronic pancreatitis: A systematic review and meta-analysis. Pancreatol: Off. J. Int. Assoc. Pancreatol (IAP) [et Al.] 25, 860–867. doi: 10.1016/j.pan.2025.08.006 40813224

[B64] Malik Z. I. Ghafoor M. U. Shah S. H. B. U. Abid J. Farooq U. Ahmad A. M. R. (2025). Unlocking iron: Nutritional origins, metabolic pathways, and systemic significance. Front. Nutr. 12, 1637316. doi: 10.3389/fnut.2025.1637316 40880741 PMC12380693

[B65] Marques L. Costa B. Pereira M. Silva A. Santos J. Saldanha L. . (2024). Advancing precision medicine: A review of innovative in silico approaches for drug development, clinical pharmacology and personalized healthcare. Pharmaceutics 16, 332. doi: 10.3390/pharmaceutics16030332 38543226 PMC10975777

[B66] Moore J. Fang J. Peterson K. Ogara M. Disario J. (2006). Jejunal feeding in chronic pancreatitis [abstract. Am. J. Gastroenterol. 101. Available online at: https://journals.lww.com/ajg/fulltext/2006/09001/jejunal_feeding_in_chronic_pancreatitis:236.236.aspx.

[B67] Naimi S. Viennois E. Gewirtz A. T. Chassaing B. (2021). Direct impact of commonly used dietary emulsifiers on human gut microbiota. Microbiome 9, 66. doi: 10.1186/s40168-020-00996-6 33752754 PMC7986288

[B68] Nasser J. Mehravar S. Pimentel M. Lim J. Mathur R. Boustany A. . (2024). Elemental diet as a therapeutic modality: A comprehensive review. Digestive Dis. Sci. 69, 3344–3360. doi: 10.1007/s10620-024-08543-1 39001958 PMC11415405

[B69] Nuzzo A. Czernichow S. Hertig A. Ledoux S. Poghosyan T. Quilliot D. . (2021). Prevention and treatment of nutritional complications after bariatric surgery. Lancet Gastroenterol. Hepatol. 6, 238–251. doi: 10.1016/S2468-1253(20)30331-9 33581762

[B70] O’Keefe S. J. D. Lee R. B. Anderson F. P. Gennings C. Abou-Assi S. Clore J. . (2003). Physiological effects of enteral and parenteral feeding on pancreaticobiliary secretion in humans. Am. J. Physiol. Gastrointestinal Liver Physiol. 284, G27–G36. doi: 10.1152/ajpgi.00155.2002 12488233

[B71] Owyang C. Logsdon C. D. (2004). New insights into neurohormonal regulation of pancreatic secretion. Gastroenterology 127, 957–969. doi: 10.1053/j.gastro.2004.05.002 15362050

[B72] Pandol S. J. (2011). “ Pancreatic secretion,” in Colloquium Series on Integrated Systems Physiology: From Molecule to Function, No. 14 (San Rafael, CA: Morgan & Claypool Life Sciences), 934–943.

[B73] Poulsen J. L. Olesen S. S. Malver L. P. Frøkjær J. B. Drewes A. M. (2013). Pain and chronic pancreatitis: A complex interplay of multiple mechanisms. World J. Gastroenterol. 19, 7282–7291. doi: 10.3748/wjg.v19.i42.7282 24259959 PMC3831210

[B74] Power C. L. McBride J. M. Drake R. L. Wu J. S. (2025). “ Anatomy-small intestine,” in Cleveland Clinic Colorectal Case Studies. Eds. Wu J. S. Inkster M. D. (Cham, Switzerland: Springer Nature Switzerland), 17–22. doi: 10.1007/978-3-031-39880-3_5

[B75] Ragins H. Levenson S. M. Signer R. Stamford W. Seifter E. (1973). Intrajejunal administration of an elemental diet at neutral pH avoids pancreatic stimulation. Am. J. Surg. 126, 606–614. doi: 10.1016/S0002-9610(73)80007-8 4200423

[B76] Reddick C. A. Greaves J. R. Flaherty J. E. Callihan L. E. Larimer C. H. Allen S. A. (2023). Choosing wisely: Enteral feeding tube selection, placement, and considerations before and beyond the procedure room. Nutr. Clin. Practice: Off. Publ. Am. Soc. For. Parenteral Enteral Nutr. 38, 216–239. doi: 10.1002/ncp.10959 36917007

[B77] Reed C. C. Fan C. Koutlas N. T. Shaheen N. J. Dellon E. S. (2017). Food elimination diets are effective for long-term treatment of adults with eosinophilic oesophagitis. Alimentary Pharmacol. Ther. 46, 836–844. doi: 10.1111/apt.14290 28877359 PMC5659358

[B78] Rezaie A. Chang B. W. de Freitas Germano J. Leite G. Mathur R. Houser K. . (2025). Effect, tolerability, and safety of exclusive palatable elemental diet in patients with intestinal microbial overgrowth. Clin. Gastroenterol. Hepatol: Off. Clin. Pract. J. Am. Gastroenterol Assoc. 23, 2306–2317.e7. doi: 10.1016/j.cgh.2025.03.002 40189034

[B79] Ridtitid W. Lehman G. A. Watkins J. L. McHenry L. Fogel E. L. Sherman S. . (2017). Short- and long-term outcomes from percutaneous endoscopic gastrostomy with jejunal extension. Surg. Endoscopy 31, 2901–2909. doi: 10.1007/s00464-016-5301-3 27796601 PMC5409872

[B80] Roberts G. P. Larraufie P. Richards P. Kay R. G. Galvin S. G. Miedzybrodzka E. L. . (2019). Comparison of human and murine enteroendocrine cells by transcriptomic and peptidomic profiling. Diabetes 68, 1062–1072. doi: 10.2337/db18-0883 30733330 PMC6477899

[B81] Robinson D. C. Rudnicki M. Tito J. M. Gold M. S. (1996). Inhibition of unstimulated exocrine pancreatic secretion by peptide YY in the rat. World J. Surg. 20, 208–214. doi: 10.1007/s002689900032 8661819

[B82] Rosendahl J. Witt H. Szmola R. Bhatia E. Ozsvári B. Landt O. . (2008). Chymotrypsin C (CTRC) variants that diminish activity or secretion are associated with chronic pancreatitis. Nat. Genet. 40, 78–82. doi: 10.1038/ng.2007.44 18059268 PMC2650829

[B83] Saltzman E. Karl J. P. (2013). Nutrient deficiencies after gastric bypass surgery. Annu. Rev. Nutr. 33, 183–203. doi: 10.1146/annurev-nutr-071812-161225 23642197

[B84] Satoh K. Ouchi M. Morita A. Kashimata M. (2019). MARCKS phosphorylation and amylase release in GLP-1-stimulated acini isolated from rat pancreas. J. Physiol. Sciences: JPS 69, 143–149. doi: 10.1007/s12576-018-0621-9 29845509 PMC10717726

[B85] Schapiro H. Britt L. G. Evans S. R. Wilson H. (1967). Effect of infusion of hypertonic fluids into the upper intestines on pancreatic secretion. Am. J. Surg. 113, 65–69. doi: 10.1016/0002-9610(67)90258-9 6016710

[B86] Schneider A. Barmada M. M. Slivka A. Martin J. A. Whitcomb D. C. (2004). Clinical characterization of patients with idiopathic chronic pancreatitis and SPINK1 mutations. Scandinavian J. Gastroenterol. 39, 903–904. doi: 10.1080/00365520410006710 15513391

[B87] Sellers Z. M. MacIsaac D. Yu H. Dehghan M. Zhang K.-Y. Bensen R. . (2018). Nationwide trends in acute and chronic pancreatitis among privately insured children and non-elderly adults in the United States 2007–2014. Gastroenterology 155, 469–478.e1. doi: 10.1053/j.gastro.2018.04.013 29660323 PMC6067969

[B88] Seto T. Grondin J. A. Khan W. I. (2025). Food additives: Emerging detrimental roles on gut health. FASEB Journal: Off. Publ. Fed. Am. Societies For. Exp. Biol. 39, e70810. doi: 10.1096/fj.202500737R 40622070 PMC12232514

[B89] Shea J. C. Bishop M. D. Parker E. M. Gelrud A. Freedman S. D. (2003). An enteral therapy containing medium-chain triglycerides and hydrolyzed peptides reduces postprandial pain associated with chronic pancreatitis. Pancreatology 3, 36–40. doi: 10.1159/000069144 12649562

[B90] Singh A. Bush N. Bhullar F. A. Faghih M. Moreau C. Mittal R. . (2023). Pancreatic duct pressure: A review of technical aspects and clinical significance. Pancreatology 23, 858–867. doi: 10.1016/j.pan.2023.09.141 37798192

[B91] Singh S. Chang H.-Y. Richards T. M. Weiner J. P. Clark J. M. Segal J. B. (2013). Glucagonlike peptide 1-based therapies and risk of hospitalization for acute pancreatitis in type 2 diabetes mellitus: A population-based matched case-control study. JAMA Intern. Med. 173, 534–539. doi: 10.1001/jamainternmed.2013.2720 23440284

[B92] Sjölund K. Sandén G. Håkanson R. Sundler F. (1983). Endocrine cells in human intestine: An immunocytochemical study. Gastroenterology 85, 1120–1130. 6194039

[B93] Skipworth J. R. A. Raptis D. A. Wijesuriya S. Puthucheary Z. Olde Damink S. W. M. Imber C. . (2011). The use of nasojejunal nutrition in patients with chronic pancreatitis. JOP: J. Pancreas 12, 574–580. doi: 10.6092/1590-8577/476 22072246

[B94] Stanga Z. Giger U. Marx A. DeLegge M. H. (2005). Effect of jejunal long-term feeding in chronic pancreatitis. J. Parenteral Enteral Nutr. 29, 12–20. doi: 10.1177/014860710502900112 15715269

[B95] Symersky T. Vu M. K. Frölich M. Biemond I. Masclee A. A. M. (2002). The effect of equicaloric medium‐chain and long‐chain triglycerides on pancreas enzyme secretion. Clin. Physiol. Funct. Imaging 22, 307–311. doi: 10.1046/j.1475-097X.2002.00435.x 12487002

[B96] Taylor C. J. Chen K. Horvath K. Hughes D. Lowe M. E. Mehta D. . (2015). ESPGHAN and NASPGHAN report on the assessment of exocrine pancreatic function and pancreatitis in children. J. Pediatr. Gastroenterol. Nutr. 61, 144–153. doi: 10.1097/MPG.0000000000000830 25915425

[B97] Thierens N. D. Verdonk R. C. Löhr J. M. van Santvoort H. C. Bouwense S. A. van Hooft J. E. (2025). Chronic pancreatitis. Lancet 404, 2605–2618. doi: 10.1016/S0140-6736(24)02187-1 39647500

[B98] Urrutia-Pereira M. Guidos Fogelbach G. Chong-Neto H. J. Solé D. (2025). Food additives and their impact on human health. Allergologia Immunopathol 53, 26–31. doi: 10.15586/aei.v53i2.1149 40088018

[B99] Vallejo C. Channagiri R. Barnes S. Vallejo K. Nazarian R. (2024). S2388 chronic pancreatitis secondary to semaglutide use. Am. J. Gastroenterol. 119, S1706–S1706. doi: 10.14309/01.ajg.0001038920.86891.ba 39449940

[B100] van Veldhuisen C. L. Kempeneers M. A. de Rijk F. E. M. Bouwense S. A. Bruno M. J. Fockens P. . (2025). Long-term outcomes of early surgery vs endoscopy first in chronic pancreatitis: Follow-up analysis of the ESCAPE randomized clinical trial. JAMA Surg. 160, 126–133. doi: 10.1001/jamasurg.2024.5182 39565607 PMC11579886

[B101] Vidon N. Hecketsweiler P. Butel J. Bernier J. J. (1978). Effect of continuous jejunal perfusion of elemental and complex nutritional solutions on pancreatic enzyme secretion in human subjects. Gut 19, 194–198. doi: 10.1136/gut.19.3.194 631640 PMC1411917

[B102] Vipperla K. Kanakis A. Slivka A. Althouse A. D. Brand R. E. Phillips A. E. . (2021). Natural course of pain in chronic pancreatitis is independent of disease duration. Pancreatology 21, 649–657. doi: 10.1016/j.pan.2021.01.020 33674197

[B103] Vu M. K. Van Der Veek P. P. Frölich M. Souverijn J. H. Biemond I. Lamers C. B. . (1999). Does jejunal feeding activate exocrine pancreatic secretion? Eur. J. Clin. Invest 29, 1053–1059. doi: 10.1046/j.1365-2362.1999.00576.x 10583454

[B104] Wan X. Bi J. Gao X. Tian F. Wang X. Li N. . (2015). Partial enteral nutrition preserves elements of gut barrier function, including innate immunity, intestinal alkaline phosphatase (IAP) level, and intestinal microbiota in mice. Nutrients 7, 6294–6312. doi: 10.3390/nu7085288 26247961 PMC4555127

[B105] Watanabe S. Shiratori K. Takeuchi T. Chey W. Y. You C. H. Chang T. M. (1986). Release of cholecystokinin and exocrine pancreatic secretion in response to an elemental diet in human subjects. Digestive Dis. Sci. 31, 919–924. doi: 10.1007/BF01303211 3731983

[B106] Wewer Albrechtsen N. J. Albrechtsen R. Bremholm L. Svendsen B. Kuhre R. E. Poulsen S. S. . (2016). Glucagon-like peptide 1 receptor signaling in acinar cells causes growth-dependent release of pancreatic enzymes. Cell Rep. 17, 2845–2856. doi: 10.1016/j.celrep.2016.11.051 27974199

[B107] White H. King L. (2014). Enteral feeding pumps: Efficacy, safety, and patient acceptability. Med. Devices 7, 291–298. doi: 10.2147/MDER.S50050 25170284 PMC4146327

[B108] Wolfe B. M. Keltner R. M. Kaminski D. L. (1975). The effect of an intraduodenal elemental diet on pancreatic secretion. Surgery Gynecol Obstetrics 140, 241–245. 1124475

[B109] Xie Y. Choi T. Al-Aly Z. (2025). Mapping the effectiveness and risks of GLP-1 receptor agonists. Nat. Med. 31, 951–962. doi: 10.1038/s41591-024-03412-w 39833406

[B110] Yadav D. Askew R. L. Palermo T. Li L. Andersen D. K. Chen M. . (2023). Association of chronic pancreatitis pain features with physical, mental, and social health. Clin. Gastroenterol. Hepatol. 21, 1781–1791.e4. doi: 10.1016/j.cgh.2022.09.026 36191836 PMC10065964

[B111] Yadav D. Timmons L. Benson J. T. Dierkhising R. A. Chari S. T. (2011). Incidence, prevalence, and survival of chronic pancreatitis: A population-based study. Am. J. Gastroenterol. 106, 2192–2199. doi: 10.1038/ajg.2011.328 21946280

[B112] Zhang G. Zhang K. Cui W. Hong Y. Zhang Z. (2018). The effect of enteral versus parenteral nutrition for critically ill patients: A systematic review and meta-analysis. J. Clin. Anesth. 51, 62–92. doi: 10.1016/j.jclinane.2018.08.008 30098572

[B113] Zhang B. Zhao C. Zhang X. Li X. Zhang Y. Liu X. . (2022). An elemental diet enriched in amino acids alters the gut microbial community and prevents colonic mucus degradation in mice with colitis. mSystems 7, e0088322. doi: 10.1128/msystems.00883-22 36468853 PMC9765100

